# ADVANCING GERONTOLOGY THROUGH EXCEPTIONAL SCHOLARSHIP (AGES): FIRST INSIGHTS FROM A NEW MENTORSHIP MODEL

**DOI:** 10.1093/geroni/igad104.3166

**Published:** 2023-12-21

**Authors:** Juanita-Dawne Bacsu, Zachary Baker, Darina Petrovsky, Zahra Rahemi, Justine Sefcik, Kris Pui Kwan Ma, Matthew Smith

**Affiliations:** Thompson Rivers University, Kamloops, British Columbia, Canada; Arizona State University, Tempe, Arizona, United States; Rutgers University, Jackson, New Jersey, United States; Clemson University, Clemson, South Carolina, United States; Drexel University College of Nursing and Health Professions, Philadelphia, Pennsylvania, United States; University of Washington, Seattle, Washington, United States; Texas A&M University, College Station, Texas, United States

## Abstract

Mentorship is vital to supporting professional growth among early career faculty members. Working with the Gerontological Society of America, the Advancing Gerontology through Exceptional Scholarship (AGES) Program was created as a mentorship model to promote productivity and peer support for early career faculty members. This presentation will: 1) describe the AGES Program as a prototype to facilitate peer support, collective learning, productivity, and co-authorship opportunities to advance early career faculty members; and 2) identify effective strategies to facilitate other groups looking to develop high-quality mentorship and training programs to support early career faculty members. After a competitive process, a cohort of 7 AGES members (including 2 program co-leads and 5 participants who all studied dementia) partook in monthly workshops from November 2022 to June 2023. Drawing on our experience with the AGES Program, we identified four strategies that cultivated our AGES Program: 1) being adaptable to address mentorship needs; 2) establishing accountability measures to enhance productivity; 3) fostering collective learning and peer support; and 4) delivering inspirational and educational activities. Following on the completion of the AGES Program, an open discussion was held with the cohort to assess and critically reflect on the program. Discussion results indicate the AGES Program was valuable in supporting early career faculty members. Specifically, findings demonstrate that the program was perceived as successfully supporting team science, collaborative opportunities, and enhancing productivity. Next steps are to conduct an anonymous evaluation survey to identify specific areas to strengthen and enhance the program for future cohorts.

